# Is Partially Thrombosed False Lumen Really a Predictor for Adverse Events in Uncomplicated Type B Aortic Dissection: A Systematic Review and Meta-Analysis?

**DOI:** 10.3389/fcvm.2021.788541

**Published:** 2022-01-18

**Authors:** Jinlin Wu, Jian Song, Xin Li, Jue Yang, Changjiang Yu, Chenyu Zhou, Tucheng Sun, Ruixin Fan

**Affiliations:** ^1^Department of Cardiac Surgery, Guangdong Cardiovascular Institute, Guangdong Provincial People's Hospital, Guangdong Academy of Medical Sciences, Guangzhou, China; ^2^Department of Vascular Surgery, Fuwai Hospital, National Center for Cardiovascular Diseases, Chinese Academy of Medical Sciences and Peking Union Medical College, Beijing, China

**Keywords:** type B aortic dissection, partial thrombosis, false lumen, meta-analysis, aortic growth

## Abstract

**Objective:**

This meta-analysis and systematic review investigated whether partial thrombosed false lumen was a predictor for adverse events in uncomplicated Type B aortic dissection (TBAD).

**Methods:**

We performed the current systematic review of the medical literature according to the 2009 Preferred Reporting Items for Systematic Reviews and Meta-Analyses (PRISMA) statement. The Newcastle-Ottawa Scale was used to evaluate the quality of individual studies. Search terms based on the MEDLINE database included “type B aortic dissection,” “false lumen” and “thrombosis.” The primary outcomes included mortality, intervention, and aortic growth.

**Results:**

Six studies were included in this systematic review, with a total number of 692 patients, including 197 patency (28.5%), 214 partial thrombosis (30.9%), and 281 complete thrombosis (40.6%). Due to the insufficient data for quantitative analysis, we only conducted a scoping review for mortality and intervention. For aortic growth, we conducted a meta-analysis based on Standardized Mean Difference (SMD). The SMD of PT vs. P by random effect model was −0.05 (random effect model) [95% confidence interval (CI), −0.39 to 0.29]. The 95% CI crossed with the null line of 0, indicating no significant difference. The SMD was 0.37 (fixed effects model) (95% CI, 0.03–0.71) and 0.70 (fixed effects model) (95% CI, 0.37–1.04) for PT vs. CT, and P vs. CT, respectively.

**Conclusions:**

Current researches on partial thrombosis of TBAD are inconsistent. Partial thrombosis is not associated with a faster aortic growth rate. Until more solid evidence is available, we do not recommend partial thrombosis as a surgical indication or high-risk profile for TBAD.

**Systematic Review Registration:** Unique Identifier: CRD42019121912.

## Introduction

Type B Aortic Dissection (TBAD) refers to dissection involving the descending aorta and was classified as complicated and uncomplicated type. According to the 2014 European Society of Cardiology (ESC) guidelines, complicated TBAD requires timely intervention to prevent dissection progression or other fatal complications, while uncomplicated TBAD can be treated conservatively with aggressive blood pressure control under close surveillance (1). Uncomplicated TBAD was defined as the absence of the following criteria: malperfusion syndrome, rupture/impending rupture, resistant hypertension, persistent pain related to TBAD, and/or rapid growth. The outcomes of uncomplicated TBAD are generally satisfactory, with up to 90% of patients surviving to discharge after effective medical treatment (2). However, uncomplicated TBAD was demonstrated to be a heterogeneous entity. Best medical treatment (BMT) may contribute to the progression of adverse events in some patients, such as shifting to a complicated type (3). The reported survival rates for conservatively treated TBAD ranged from 56 to 92%, and 48 to 82% at 1 year, and 5 years, respectively (4, 5). On the other hand, patients with uncomplicated TBAD may not obtain additional benefits from thoracic endovascular aortic repair (TEVAR) but be exposed to the side effects of TEVAR such as leakage, stent displacement, and retrograde dissection, et al. As shown by the INSTEAD trial, the first randomized study on elective stent-graft placement in survivors of uncomplicated type B aortic dissection, TEVAR failed to improve 2-year survival and adverse event rates despite favorable aortic remodeling compared with optimal medical therapy (6). The prognosis of uncomplicated TBAD seems elusive. Therefore, it is important to stratify the risk of uncomplicated TBAD so that we can timely intervene in high-risk groups and improve the overall survival rate. Among other factors (7–10), aortic morphology is a classic and reliable predictor, such as diameter, location, and the number of entry tears. The thrombosis status of the false lumen is gaining more attention recently.

Intuitively, we may expect complete thrombosis to have the best outcome, followed by partial thrombosis and patent false lumen. It is universally accepted that complete thrombosis is associated with a better prognosis than that of a patent false lumen (11, 12). However, the role of partial thrombosis (concurrent presence of both flow and thrombus) remains controversial, which was first proposed by Tsai et al. in 2007 (13). They found that partial thrombosis at discharge was a strong predictor of mortality in patients with TBAD compared with complete thrombosis and patency. They reported that the mean 3-year mortality rate for patients with a patent false lumen was 13.7 ± 7.1%, for those with partial thrombosis was 31.6% ± 12.4, and for those with complete thrombosis was 22.6% ± 22.6% (*p* = 0.003).

There is a heated debate as to whether partially thrombosed false lumen was really a predictor for adverse events in uncomplicated TBAD. Therefore, we carried out the current systematic review and meta-analysis.

## Methods

This study has been pre-registered, and we published the protocol at the International Prospective Register of Systematic Reviews before study commencement (registration number: CRD42019121912). We carried out the current systematic review of the medical literature according to the 2009 Preferred Reporting Items for Systematic Reviews and Meta-Analyses (PRISMA) statement guidelines (14).

### Literature Search Strategy

**Figure 1:**
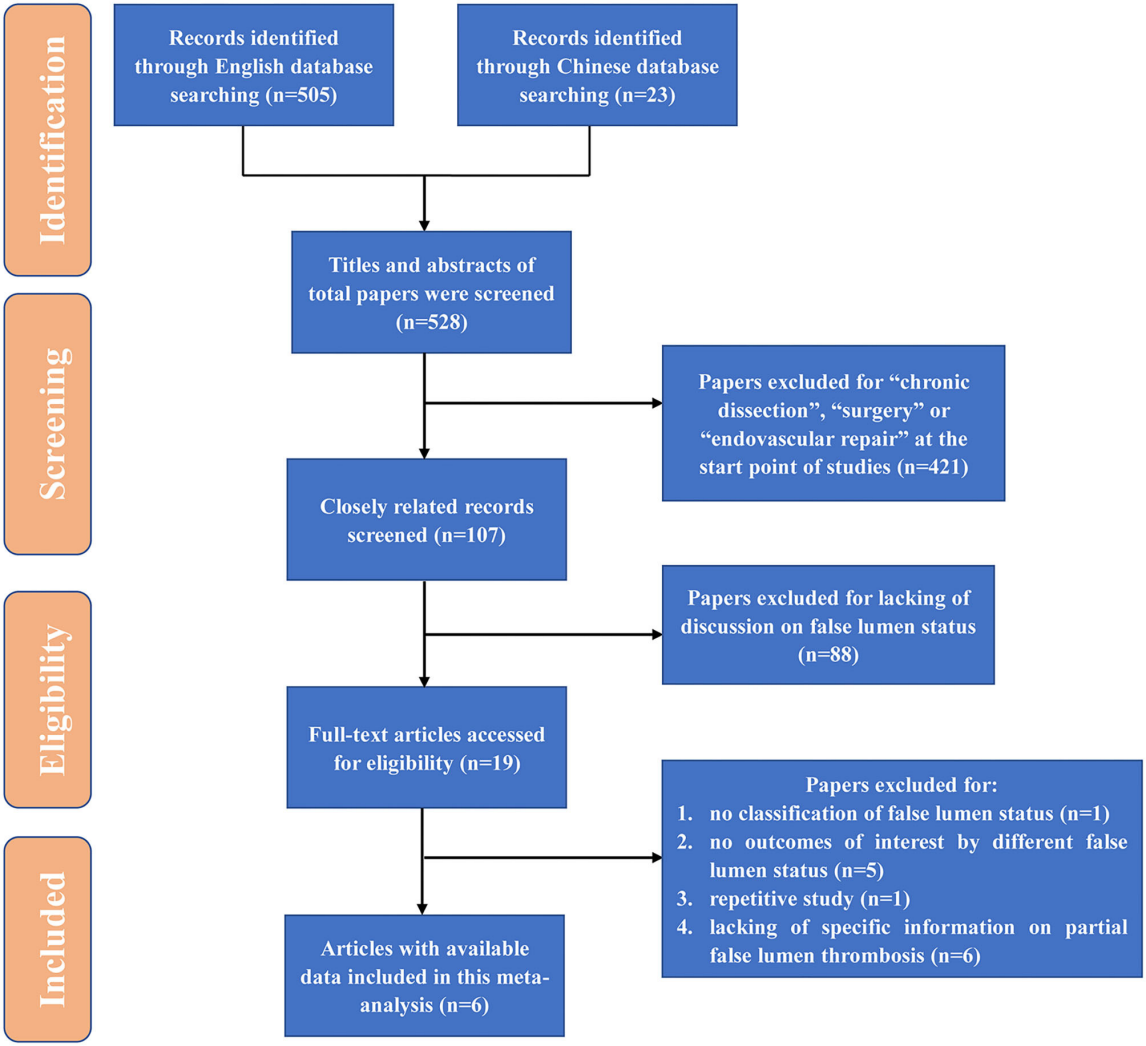
Flow diagram for study identification. TEVAR, thoracic endovascular aortic repair; HR, hazard ratio; OR, odds ratio.

All studies regarding partial thrombosis in the setting of medically treated TBAD were recognized through a two-step search approach. The first search was done on PubMed from its commencement to June 2021. Next, relevant studies were recognized through manual examination of secondary sources comprising references of formerly identified articles and a search of reviews and commentaries. All relevant publications were downloaded for confirmation and further analysis, and duplicates were excluded. Search terms included “type B aortic dissection” “false lumen” “thrombosis.” We included studies as follows: (1) uncomplicated TBAD; (2) classification and definition of different false lumen status. Those studies that involve surgery, TEVAR, and did not report partial thrombosis were excluded. Studies were also excluded from particular pooled outcome estimates if not reporting the specific outcome measure. Conference abstracts, case reports, editorials, expert opinions, and reviews were also excluded. In cases where centers reported outcomes of overlapping patient series, only the most contemporary series were analyzed. Through the above searching and inclusion process, we finally obtained 6 high-quality literatures (Figure 1).

### Data Extraction and Quality Assessment

**Figure 2:**
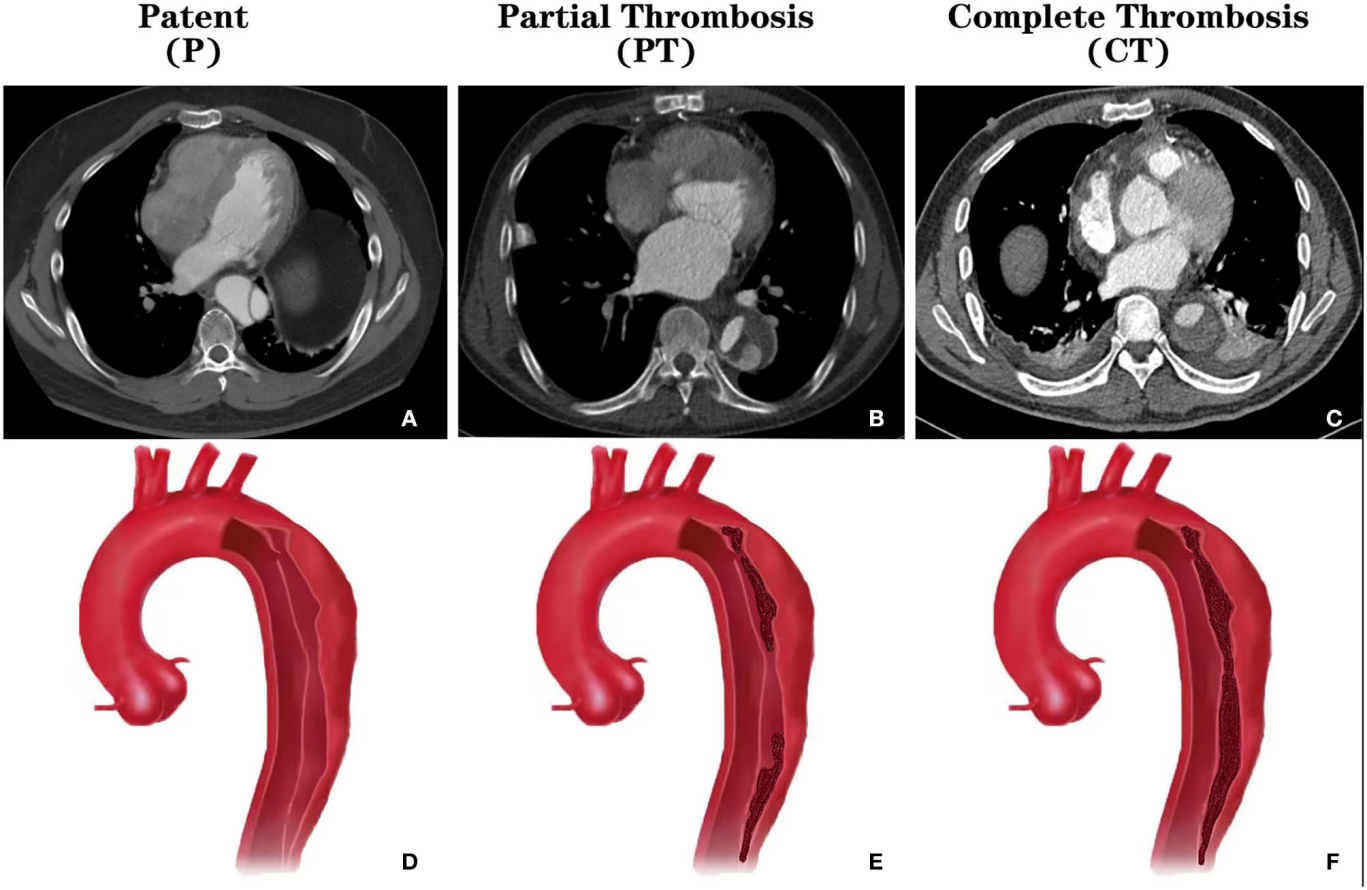
CT images and schematics of different thrombosis status of the false lumen in type B aortic dissection. **(A)** Cross-sectional CT showing the patent false lumen; **(B)** Cross-sectional CT showing the partially thrombosed false lumen; **(C)** Cross-sectional CT showing the completely thrombosed false lumen; **(D)** Schema showing the patent false lumen; **(E)** Schema showing the partially thrombosed false lumen; **(F)** Schema showing the completely thrombosed false lumen. CT, computed tomography.

Two investigators (JW and JS) extracted data, which was independently substantiated by a third investigator (RF). Divergences were set by agreement in a core meeting. TBAD was defined as any non-traumatic dissection not involving the ascending aorta and presenting within 14 days of onset. Patent false lumen (Group P) was defined by the absence of any thrombus; Partial thrombosis (Group PT) was defined as circulatory flow despite the presence of a thrombus; Complete thrombosis (Group CT) was defined by the absence of any circulation at all in the false lumen (Figure 2). Primary outcomes included mortality, intervention, and growth rate. Data regarding aortic events (mortality and intervention) were extracted in three ways: directly extract from the article, contact the author via email, and roughly estimate through the Kaplan-Meier curve. Standard deviation (SD) of the growth rate was not reported in a study, data imputation was performed with the mean of other studies. The Newcastle-Ottawa Scale (NOS) was used to evaluate the quality of individual studies (15). A final score higher than six was considered as high quality.

### Statistical Analysis

To summarize the demographical and baseline data of the recruited patients from all eligible published studies, continuous variables were reported as mean with standard deviation (SD), and categorical variables were reported as a number with the percentage. Heterogeneity between studies was analyzed utilizing the Q test and *I*^2^ index. The extent of variation among the effects observed in different studies (inter-study variance) is referred to as τ^2^. Given that we did not extract enough data for quantitative analysis, we could only conduct a scoping review for mortality and intervention (12). For aortic growth, we conducted a meta-analysis based on the standardized mean difference (SMD). A fixed-effects model was preferred, while a random-effects model was used if high heterogeneity existed among studies.

All analyses were conducted using R software (version 3.6.1). A two-tailed *p* < 0.05 indicated statistical significance.

## Results

### Search Results and Study Characteristics

The initial databases search yielded 528 potential publications. The literature screening process was shown in Figure 1. Eventually, 6 studies (16–21) were included in this systematic review, with a total number of 692 patients, including 197 patency (28.5%), 214 partial thrombosis (30.9%), and 281 complete thrombosis (40.6%). Characteristics of included studies are shown in Table 1. The studies included were cohort studies. The average follow-up duration was from 1.6 to 5.1 years. The NOS scores indicated a low risk of bias. Most studies were from Japan (50%, 3/6). All of the included studies were published in the leading journals of cardiovascular surgery, including the Journal of Thoracic and Cardiovascular Surgery (50%, 3/6) and Annals of Thoracic Surgery (50%, 3/6). The mean age (±SD) of the 528 patients ranged from 60.3 ± 10.7 to 70.4 ± 11.8, and 66.1–69.4% were male, and 70.0–82.1% had a history of hypertension. The prevalence of Marfan syndrome was low, which was reported by two studies of 0.9 and 2.6%, respectively.

**Table 1:** Characteristics of the included studies.

**First author, reference**	**Nation**	**Journal**	**Design**	**Study period**	**Median/mean follow-up (years)**	**Total sample**	**Age (Mean ± SD)**	**Male (n, %)**	**Hypertension (*n*, %)**	**Marfan's Syndrome (*n*, %)**	**NOS score**
Chikara Ueki (21)	Japan	Annals of Thoracic Surgery	Cohort study	2003–2012	3.2	228	70.4 ± 11.8	153, 67.1%	/	2, 0.9%	6
Tomoaki Kudo (20)	Japan	Journal of Thoracic and Cardiovascular Surgery	Cohort study	1991–2012	5.1	95	68.9 ± 11.3	66, 69.4%	78, 82.1%	/	7
Santi Trimarchi (18)	Multination	Journal of Thoracic and Cardiovascular Surgery	Cohort study	1998–2011	1.6	84	/	/	/	/	6
Jip L. Tolenaar (19)	Multination	Journal of Vascular Surgery	Cohort study	2000–2010	1.9	62	60.3 ± 10.7	41, 66.1%	/	/	7
Frederik H. W. Jonker (17)	Multination (IRAD)	Annals of Thoracic Surgery	Cohort study	1996–2010	2	191	62.1 ± 13.0	132, 69.1%	133, 70.0%	1, 2.6%	7
Eijun Sueyoshi (16)	Japan	Annals of Thoracic Surgery	Cohort study	1997–2007	4.1	71	64.4 ± 11.9	49, 69.0%	71, 74.6%	/	7

*NOS, Newcastle-Ottawa Scale; IRAD, the international registry of acute aortic dissection; SD, standard deviations*.

### Mortality

There was no consensus as to whether partial thrombosis would trigger a higher mortality risk for uncomplicated TBAD. According to Ueki et al. (*n* = 228) (21), survival was lowest in group PT, with 1-, 3-, and 5-year survival rates of 85.5% ± 5.5%, 81.2% ± 6.7%, and 75.0% ± 8.6%, respectively vs. 92.6% ± 5.0%, 86.8% ± 7.3%, and 86.8% ± 7.3% in group P, and 97.6% ± 1.4%, 91.2% ± 3.1%, and 85.9% ± 4.2% in group C. Separate log-rank testing revealed a significant increase in mortality in group PT compared with group C (p=0.013). There was no significant difference between groups P and C or between groups P and PT. Kudo et al. (*n* = 95) (20) reported that the 5-year survival rate for groups P, PT, and CT were 88, 88, 87.1%, respectively. And the 10-year survival rate for groups P, PT, and CT were 64.5, 78.2, 55.3%, respectively. There were no significant differences between the three groups.

### Intervention

Sueyoshi et al. (*n* = 71) (16) reported that the freedom from aortic repair rates at 1, 2, 5, and 10 years were 88, 78, 61, and 61% for group P, respectively; 85, 79, 68, and 59% for group PT; and 100, 100, 75, and 75% for group CT. According to the Kaplan-Meier curve, group CT had the highest survival rate, but there was no significant difference between groups PT and P.

### Growth Rate

**Figure 3:**
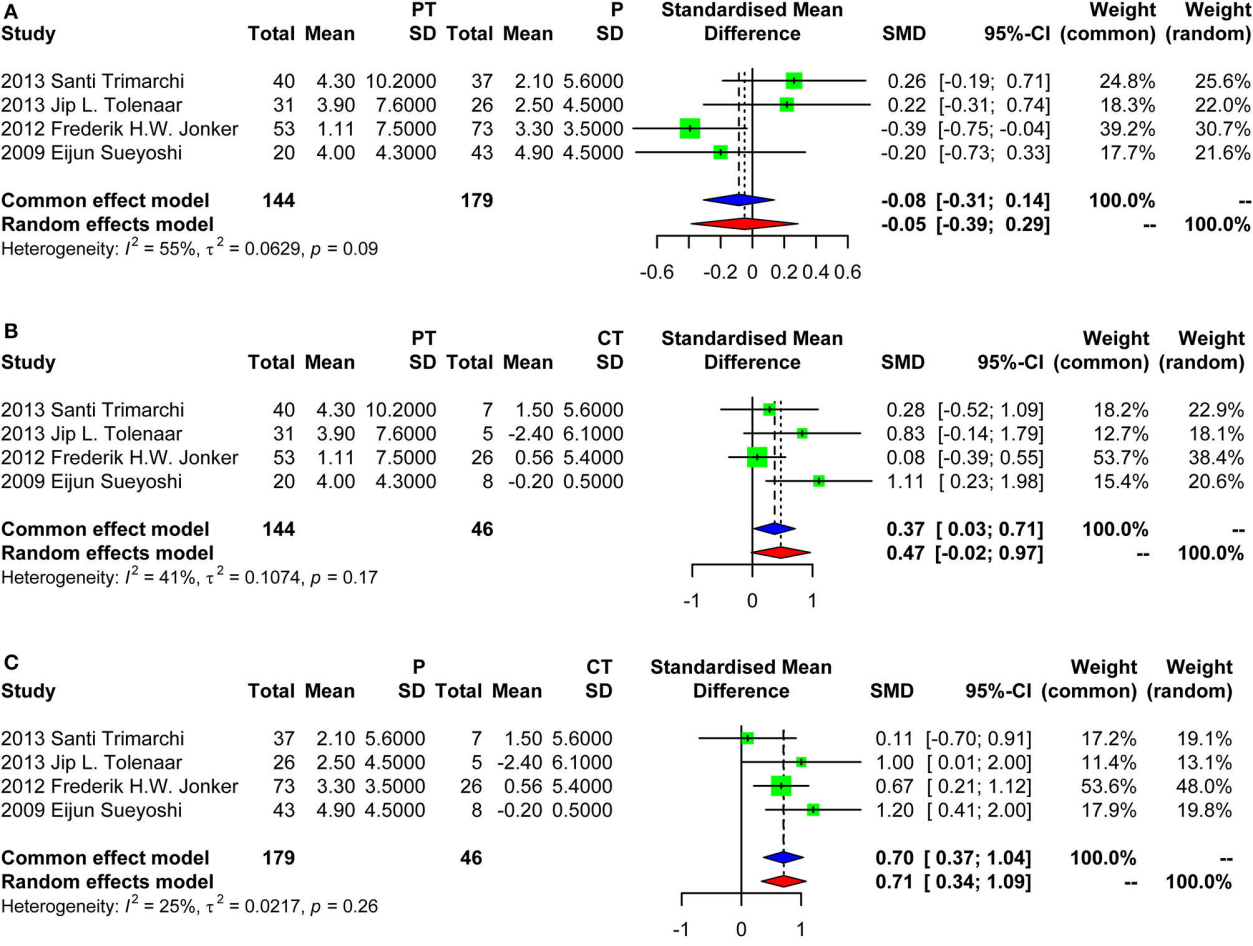
Forest plot showing the meta-analysis of aortic growth. **(A)** Group PT vs. group P, with a pooled SD of −0.05 (random effect model) [95% confidence interval (CI), −0.39 to 0.29]; **(B)** Group PT vs. group CT, with a pooled SD of 0.37 (fixed effects model) (95% CI, 0.03–0.71); **(C)** Group P vs. group CT, with a pooled SD of 0.70 (fixed effects model) (95% CI, 0.37–1.04). PT, partial thrombosis; P, patency; CT, complete thrombosis; SD, standard deviation; CI, confidence interval.

**Figure 4:**
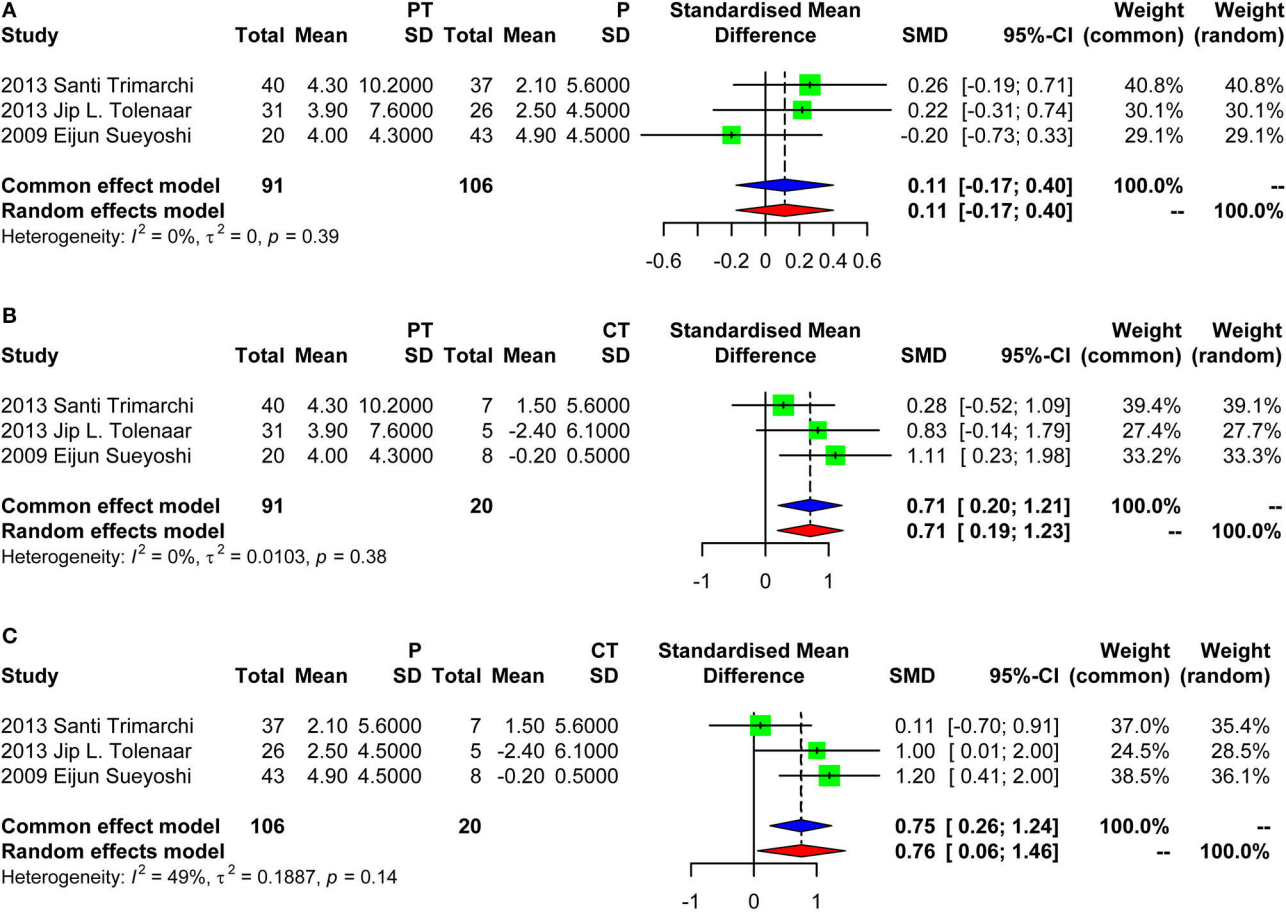
Forest plot showing the meta-analysis of aortic growth excluding the study of Frederik H. W. Jonker et al. **(A)** Group PT vs. group P, with a pooled SD of 0.11 (fixed effect model) [95% confidence interval (CI), −0.17 to 0.40]; **(B)** Group PT vs. group CT, with a pooled SD of 0.71 (fixed effects model) (95% CI, 0.20–1.21); **(C)** Group P vs. group CT, with a pooled SD of 0.75 (fixed effects model) (95% CI, 0.26–1.24). PT, partial thrombosis; P, patency; CT, complete thrombosis; SD, standard deviation; CI, confidence interval.

Four studies were included in the meta-analysis of aortic growth as shown in Figure 3. The SMD of group PT vs. group P was −0.05 (random effect model) [95% confidence interval (CI), −0.39 to 0.29]. The 95% CI crossed the null line of 0, indicating no significant difference. The SMD of group PT vs. group CT was 0.37 (fixed effects model) (95% CI, 0.03–0.71). The SMD of group P vs. group CT was 0.70 (fixed effect model) (95% CI, 0.37–1.04). Those data indicated that group PT was not associated with a faster growth compared with group P. Group CT had the lowest growth rate as expected. Of note, the SD of the aortic growth in the study by Frederik H. W. Jonker et al. was imputed with the average of the SDs of all other included studies. We performed a sensitivity analysis further by excluding the study of Frederik H. W. Jonker et al. as demonstrated in Figure 4. The results remained quite stable, which further consolidated the above findings.

## Discussion

Early studies generally classified the false lumen in a dichotomy way: patency and complete thrombosis. The effect of partial thrombosis on the prognosis of TBAD was firstly proposed by Tsai et al. (13), inspiring subsequent researches. The six studies we included were all published after that. Tsai et al. found that the risk of death is increased by a factor of 2.7 among patients with partial thrombosis of the false lumen compared with patients who have completely patent false lumens (no thrombus). Although not proven, this study suggested two mechanisms by which partial thrombosis may lead to adverse outcomes: hemodynamic and hypoxia/inflammatory mechanism. In the first theory, thrombosis at the distal end of the false lumen may block the secondary entry tear to form a “blind sac” structure, increasing the pressure of the false lumen (22). In the second theory, partial thrombosis may increase the risk of rupture as a result of hypoxia of the arterial wall adjacent to the intraluminal thrombus, which leads to increased local inflammation, neovascularization, and localized wall weakening (23). Of note, in the study by Tsai et al. some patients were treated with surgery or TEVAR, which is a source of clinical heterogeneity. We, therefore, excluded it from this meta-analysis. And its endpoint was all-cause death instead of aortic-related death. Kudo et al. followed up 117 patients with uncomplicated TBAD for 5.1 ± 4.1 years (range, 0.1–20.1), and found that (20) 95 survived and 22 died, including the 3 with aortic rupture. The other 19 deaths were not caused by aortic events; 6 died of cancer, 7 of heart failure, and 6 of weakness. In other words, most of the deaths were not related to aortic events. Kudo et al. also found no difference in the survival rate among groups PT, P, and CT. The event-free rate was the greatest in group CT, with a 3- and 5-year event-free rate of 100 and 95.7%, respectively. The log-rank test showed that the event-free rate in group T was significantly higher than that in the other groups (group CT vs. group P, *p* < 0.0001; group CT vs. group PT, *p* = 0.0009).

As discussed above, it remains hugely controversial of the role of partial thrombosis. In particular, the sample size and the number of event of previous studies were small, which leads to low statistical power and uncertain conclusions. Apart from aortic events, aortic growth was demonstrated to be highly correlated with aneurysm formation and rupture. Our study found group P was not associated with a faster growth compared with group P and group CT had the lowest growth rate as expected. Sueyoshi et al. (16) firstly studied whether a partially closed false lumen affects aortic enlargement in patients with TBAD in 2009. They found that aortic growth rates for groups CT, PT, and P were −0.2 ± 0.5, 4.0 ± 4.3, and 4.9 ± 4.5 mm/year, respectively (*p* = 0.0149). Consistent with us, the found that partial thrombosis was not a risk factor for aortic enlargement. Interestingly, they pointed out that the sac formation PT type was the culprit, which was defined as a partially closed false lumen in the distal portion of the entry site of the false lumen. Remarkably, in patients with partial thrombosis, the growth rates in the sac and non-sac groups were 12.7 ± 1.1 and 2.6 ± 2.7 mm/year, respectively (*p* = 0.007). This phenomenon partly confirmed the hemodynamic mechanism inferred by Tsai et al. However, sac formation type was found in only three patients (15%) out of group PT (*N* = 20). This should not raise the risk level of group PT as a whole, nor did it explain the greater risk of group PT as shown by Tsai et al. A subsequent IRAD study (17) also confirmed the findings of Eijun Sueyoshi et al. They reported that group PT had not been observed to grow faster: Group P was the fastest (3.31 mm/year), followed by group PT (1.11 mm/year), and group CT (0.56 mm/year). The study by Trimarchi et al. contradicted the above findings (18). Their results showed that partial thrombosis of the false lumen (vs. patent false lumen) had a significant effect on the annual aortic growth rate (*p* < 0.05 in all). The annual aortic expansion was significantly larger in patients with a partially thrombosed false lumen compared with a patent false lumen (*p* = 0.035). There was no significant difference between the annual aortic growth rates of dissections in patients with a partially thrombosed false lumen and patients with a completely thrombosed false lumen (*p* = 0.745). Of note, in this study, the mean aortic growth of group PT was 4.2, with a standard deviation (SD) of 10.8 (far greater than the mean), which implies greatly varied aortic growth. This kind of data is not normally distributed, and the mean could be easily affected by extreme values. The median is considered to be a more reasonable statistical method. Subsequent studies by Tolenaar et al. (19) further showed that sac formation type PT had a faster growth rate: the mean growth rate of patients with a saccular formation of the partially thrombosed FL was 7.8 ± 13.6 mm/year, which was significantly larger compared with patients with non-saccular partial lumen thrombosis (3.0 ± 5.0 mm/year; *p* = 0.007). Their data also showed that sac formation type PT accounted for only 6/31 (19.4%) of group PT. Apart from sac formation, the volume of the thrombosis and the communications between the false lumen and aortic branches were also important confounding variables, deserving further investigations.

Almost all the studies calculated the growth rate with the last size-first size/time difference. This method assumed that aortic growth was linear and lost the information of intermediate measurements. The instrumental variable approach (24) or the mixed-effects model (25) would be more reasonable. In addition, there are mixed views on whether aortic segmentation should be performed. Some studies evaluated the entire aorta collectively, while others evaluated the dissection based on different segments. For example, a patient would be classified as partial thrombosis collectively if he had a patent upper segment and a completely thrombosed lower segment (19). Another issue is that some studies excluded intramural hematoma (IMH), while others took IMH as group CT. These issues need to be standardized and clearly stated in subsequent studies so that different studies can be compared and meaningful conclusions can be drawn.

Although somewhat beyond the scope of this research, it has to be pointed out that identification of high-risk uncomplicated TBAD requires a comprehensive assessment based on multiple indicators including clinical manifestations, biomarkers, diameter, growth rate, ulcer-like projection(ULP), thickness of false lumen, location and the size of entry tears, rather than a single indicator such as thrombosis status. Tolenaar et al. studied (19) multiple morphologic characteristics appearing to predict aortic dilatation in TBAD patients treated medically, and found that a saccular formation of the FL, the number of intimal tears, the location of the intimal tear, and configuration of the TL may influence aortic growth rate during follow-up. A “nomogram,” an easy-to-use multivariate bedside tool, may be considered by future studies to develop personalized prediction model (26).

An important issue that has not been investigated by previous studies is the evolution of false lumen thrombosis, and its effect on clinical outcomes. According to our clinical experience, a patent false lumen may evolve into partial thrombosis and then complete thrombosis, or vice versa (27). Compared to the information obtained from a single CT measurement, it may be more comprehensive and reliable to investigate the false lumen status shifting, its duration and the influencing factors. As shown by Trimarchi et al. (18), out of 84 patients, of whom 40 (47.6%) had a partially thrombosed false lumen, 7 (8.3%) had a completely thrombosed false lumen, and 37 (44.0%) had a patent false lumen. During follow-up, false lumen status changed from patent to partial thrombosis in 8 patients, partial thrombosis to patent false lumen in 2 patients, and partial to complete thrombosis in 4 patients. Kudo et al. (20) also noted some patients experienced shifts in the lumen status. False lumen status may change not only during follow-up, but even during hospitalization. Tanaka et al. reported that (28) false lumen changed from complete thrombosis to partial thrombosis in three patients, partial thrombosis to complete thrombosis in four patients, and patent to partial thrombosis in two patients during initial hospitalization. It is quite astonishing if we consider that CT images taken at different time points would certainly influence the grouping of false lumen status. This may partly explain the substantial outcome variations between different studies. Another issue that needs to be clarified is whether it is appropriate to continue to analyze according to the original grouping after the lumen status has changed.

### Study Limitations

The findings of this meta-analysis should be interpreted in the background of several limitations. First, even though many studies enrolled in this meta-analysis were of high quality, the retrospective and observational nature of the investigated data raise the risk of bias. Second, the small sample size of individual studies may result in insufficient statistical power to detect differences in outcome between different false lumen status.

## Conclusion

The researches on partial thrombosis of uncomplicated TBAD are inconsistent. In view of the prevalent confounding factors and small sample sizes for current researches, we do not recommend partial thrombosis as a surgical indication until more solid evidence is available. The sac formation type of partial thrombosis seems an alerting sign worthy of further investigations. Several issues need to be clarified for future studies such as the sample size determination, aortic-related death, standardized CT measurement including the time point of the image taking, status shifting, multivariable prediction model, segmentation, and plausible aortic growth calculation, etc.

## Data Availability Statement

The original contributions presented in the study are included in the article/supplementary material, further inquiries can be directed to the corresponding author/s.

## Author Contributions

JW and JS: conception and design. XL: analysis and interpretation. JW and JY: data collection. JW and CY: writing the article. CZ: critical revision of the article. TS: final approval of the article. JW: statistical analysis and overall responsibility. TS and RF: obtained funding. All authors contributed to the article and approved the submitted version.

## Funding

This work was supported by the National Key Research and Development Program of China (2017YFC1308003), National Key Research and Development Program of China (2018YFC1002600), and Guangzhou Science and Technology Program Key Projects (202002020037).

## Conflict of Interest

The authors declare that the research was conducted in the absence of any commercial or financial relationships that could be construed as a potential conflict of interest.

## Publisher's Note

All claims expressed in this article are solely those of the authors and do not necessarily represent those of their affiliated organizations, or those of the publisher, the editors and the reviewers. Any product that may be evaluated in this article, or claim that may be made by its manufacturer, is not guaranteed or endorsed by the publisher.
